# Long-term stability of over-the-counter cuffless blood pressure monitors: a proposal

**DOI:** 10.1007/s12553-023-00726-6

**Published:** 2023-01-23

**Authors:** Toshiyo Tamura, Shigeru Shimizu, Nobuhiro Nishimura, Masachika Takeuchi

**Affiliations:** 1 Future Robotics Organization, Waseda University, Tokyo, Japan; 2 San-ei Medisys Co., Ltd, Kyoto, Japan

**Keywords:** Cuffless blood pressure, Regulation, Long-term stability, Auscultation sphygmomanometer, Oscillometric sphygmomanometer

## Abstract

Blood pressure is an important cardiovascular parameter. Currently, the cuff-based sphygmomanometer is a popular, reliable, measurement method, but blood pressure monitors without cuffs have become popular and are now available without a prescription. Blood pressure monitors must be approved by regulatory authorities. Current cuffless blood pressure (CL-BP) monitors are not suitable for at-home management and prevention of hypertension. This paper proposes simple criteria for over-the-counter CL-BP monitoring. First, the history of the sphygmomanometer and current standard blood pressure protocol are reviewed. The main components of CL-BP monitoring are accuracy during the resting condition, accuracy during dynamic blood pressure changes, and long-term stability. In this proposal we recommend intermittent measurement to ensure that active measurement accuracy mirrors resting condition accuracy. A new experimental protocol is proposed to maintain long-term stability. A medically approved automated sphygmomanometer was used as the standard device in this study. The long-term accuracy of the test device is based on the definition of propagation error, i.e., for an oscillometric automated sphygmomanometer (5 ± 8 mmHg) ± the error for the test device static accuracy (–0.12 ± 5.49 mmHg for systolic blood pressure and − 1.17 ± 5.06 mmHg for diastolic blood pressure). Thus, the long-term stabilities were − 3.38 ± 7.1 mmHg and − 1.38 ± 5.4 mmHg, which satisfied propagation error. Further research and discussion are necessary to create standards for use by manufacturers; such standards should be readily evaluated and ensure high-quality evidence.

**Supplementary information:**

The online version contains supplementary material available at 10.1007/s12553-023-00726-6.

## Introduction

Blood pressure (BP) is an important measure of health. It is created by the force of the blood pushing against the walls of blood vessels after the heart pumps. The heart must pump harder under conditions of high BP (hypertension). Hypertension is a serious health condition and increases the risks of heart, brain, kidney, and other diseases. It is a major cause of premature death worldwide and is present in more than one in four men and one in five women, or more than one billion people worldwide. The burden of hypertension is felt disproportionately in low- and middle-income countries, where two-thirds of cases occur, largely due to increased risk factors in those populations in recent decades [1].

Booth published a valuable review on the history of monitoring BP [2, 3]. In 1727, Hales first measured the pressure in parts of the circulation. In 1847, Lundwig developed the kymograph, the first device that enabled continuous BP recording. In 1855, an indirect, non-invasive technique was developed to measure the pulsation in an artery: after several researchers attempted to use a sphygmomanometer clinically, Riva-Rocci reported the method on which the present technique is based. His technique involved compressing the arm with a rubber bag and inflating it with air using an attached rubber bulb. The cuff pressure was registered using a mercury manometer, and was increased until the radial pulse could no longer be palpated. When the pressure was slowly released, the mercury level in the manometer fell, and the reading at which the pulse reappeared was taken as the systolic BP. In 1905, Korotkoff reported that by placing a stethoscope over the brachial artery at the cubital fossa, the systolic and diastolic pressures were determined. This auscultatory technique became a popular standard method for monitoring BP. The error of the BP value was related to the pulse oscillation. However, oscillometric BP monitors were developed in the 1980s. In clinical practice, auscultation methods were the standard. Then, automatic sphygmomanometers were developed with a microphone in a cuff and oscillometric devices followed. Subsequently, arterial tonometry and the volume clamp method were developed and applied to noninvasive continuous BP measurement. In the last two decades, additional efforts have been made to transform unobtrusive BP monitoring without using a cuff.

To monitor hypertension, a simple, easy-to-use device is preferable, and a cuffless sphygmomanometer is one such device. Due to the increasing popularity of cuffless devices, their safety and reliability are important. Post-market surveillance measures including regulatory inspections and regular calibration are warranted. The frequency of inspections and calibrations should meet legislative and regulatory requirements, and the manufacturer’s recommendations. Maintenance recommendations vary depending on device type, and the frequency and location of use. The following references outline procedures for medical inspections [4–6]; we also briefly describe these procedures in the Discussion.

Various cuffless BP (CL-BP) monitors have been developed, as reviewed elsewhere [7–17]. Section 2.1 provides a detailed explanation of CL-BP monitors. BP estimation using commercially available devices with and without medical approval has been described in published studies. Although the U.S. Food and Drug Administration (FDA) has approved a few devices, no standard has been established. Furthermore, the International Organization for Standardization (ISO) has not officially discussed these devices. Recently, the ISO published a draft of ISO/FDIS 81060-3 Noninvasive Sphygmomanometers-Part 3: Clinical Investigation of the Continuous Automated Measurement Type [18]. This standard includes cuff-based technology, mainly based on the volume clamp method. However, approval by the FDA follows ISO 81060-2 + 2018, the standard for cuff-based automated sphygmomanometers [19]. The static accuracy of CL-BP monitors approved by the FDA is based on the same ISO 81060-2 criterion. No details of dynamic BP changes or long-term stability have been published. The Institute of Electrical and Electronics Engineers (IEEE) Standards Association published a standard for wearable CL-BP measuring devices (IEEE std 1708;2014, 1708a;2019), which includes information concerning the accuracy of BP at rest and BP changes in individuals [20].

The International Organization of Legal Metrology (OIML) recently published two recommendations, for non-invasive non-automated sphygmomanometers R 148 [21] and non-invasive automated sphygmomanometers R149 [22]. The OIML and ISO regulations are clear; he primary inspections are the same, although the OIML includes some inspections that are absent from the ISO regulations. The ISO inspection statements are in ISO81060-2:30:2018 [23].

CL-BP monitors appear to be an alternative to cuff-based sphygmomanometers because they are easy to operate, inexpensive, and may not require calibration. The next section presents existing commercial devices with and without evidence studies. The discussion focuses on the reliability of CL-BP values during dynamic BP changes and their long-term stability, as well as clinical applications of CL-BP monitors. Over-the-counter CL-BP devices have become popular and are available without a prescription. Here, we review the issues surrounding home-use CL-BP monitors in terms of accuracy at rest, dynamic response, and long-term use.

Accuracy at rest follows the cuff-based sphygmomanometer under ISO 81060-2 [10, 11, 16, 17]. Regarding dynamic BP changes, CL-BP monitors conduct either continuous beat-to-beat or intermittent measurements. If a device is used for continuous BP monitoring, beat-to-beat determination must be evaluated by a standard sphygmomanometer. Although some offset is expected, only a direct BP monitor can be used as the “gold standard.” Unfortunately, some countries, such as Japan, do not allow the use of direct BP monitoring in healthy individuals for ethical reasons, so we consider a new way to estimate dynamic BP changes.

The requirement for a stable BP hampers the tracking of changes, particularly changes that occur over a few minutes; this hindrance is difficult to overcome because BP is typically unstable immediately after a change in BP. There are no reports on this topic. Therefore, we did not consider CL-BP monitors used as beat-to-beat monitors. In this report, we focus on the intermittent BP device as an alternative use of cuff-based sphygmomanometers and consider only the long-term stability. Furthermore, the initialization of BP measurement is important; for home use, long-term stability and reinitialization determination merit discussion. Thus, a simple protocol is proposed.

## Current progress in CL-BP monitors

### Manufacturing

Multiple manufacturers produce CL-BP devices based on various technologies, as listed in Table 1. Current CL-BP monitoring technologies are based on photoplethysmography( PPG) waveform, pulse transit time (i.e., PPG plus other physiological parameters), pressure sensors, tonometry, and radar. Devices based on ultrasound, impedance, and capacitance have been reported but not yet commercialized.

A brief explanation of the principle is as follows [11]. The pulse contour and acceleration methods enable the estimation of BP with only one PPG signal. Pulse demodulation analysis (PDA) was developed to evaluate the arterial pressure pulse, and pressure pulse reflections in the central and peripheral arterial tree. PTT-based BP devices are based either on PPG and electrocardiography (ECG; R wave), or on two PPGs.,  1. Continuous, non-invasive indirect BP measurement is based on changes in the pulse wave velocity (PWV), which is the velocity with which the pressure pulse propagates along the arterial wall. This can be calculated from the pulse transit time (PTT), i.e., the time between two pulse waves propagating from two separate arterial sites during the same cardiac cycle. Pressure waveform analysis also uses PDA. According to the principle of applanation tonometry of the radial artery, when the radial artery is partially compressed against bone, the pulsations are proportional to the intra-arterial pressure. Moreover a millimeter-wave sensor is attached to the forearm and estimates the BP from a single pulse signal.

**Table 1:** Commercial cuffless blood pressure monitors

Method	Measurement site	Product	References
PPG waveform	Wrist Ear or finger	Aktiia** Bioheat*,** Samsung*^3^ Deepmedi*^3^ Valencell	[24–27] [28, 29] [30]
PPG + other physiological parameters	Finger Wrist	Sotera* Somnotouch** InBddy FreeScale**	[31] [32] [24, 33]
Pressure waveform	Finger	Care Taker*	[34–36]
Tonometry	Wrist	BPro* Omron	[37, 38] [39–41]
Radar	Wrist	Blumio	[42–44]

* FDA approved device, ** CE marked, *^3^ approved in South Korea

### Clinical prospective

Hypertension is a critical diagnosis [45]. The Lancet Commission on Hypertension group reported that there was a lack of regulatory requirements for mandatory independent variation in BP devices [46]. A response suggested that there is an urgent need to create standards for CL-BP devices [47, 48]. Numerous suggestions and proposals have been published [49–52]; a simple, low-cost, highly accurate sphygmomanometer is required.

### Current research topics related to CL-BP monitors

CL-BP monitors have been evaluated in physiological studies and clinical trials. Physiological studies have investigated the reliability of the pulse transit time (PTT) at different measurement sites, such as the neck, fingers, and toes. Moreover, the relationship between BP and blood volume (in terms of blood vessel stiffness) has been evaluated [53–58]. These studies have demonstrated the limitations of PTT Even though, several clinical trials are ongoing.

The establishment of a standard is important for ensuring the reliability of CL-BP monitors, but only the IEEE is actively engaged. However, the IEEE standards are only partly recognized by the FDA [59]. Although the dynamic change in BP and long-term stability are important issues in continuous noninvasive BP monitors, few reports have been published about this.

## Long-term stability

### Overview

The proposal for long-term stability involves a two-step evaluation. First, accuracy at the resting stage is obtained by ISO81060-2, using the auscultation method. The obtained static accuracy is used to evaluate the long-term stability. Approved medical automatic oscillometric devices are used as gold standard devices for the evaluation because of their ease of handling and convenience for manufacturers. Long-term stability can be obtained as the error propagation consisting of the static accuracy of the device under test plus the static accuracy of the oscillometric BP device.

### Proposal

#### Step 1: accuracy test

The protocol for assessing accuracy at rest followed ISO 81060-2:2018 and is summarized below. CL-BP devices need to initialize using systolic and diastolic BPs obtained with a standard auscultation sphygmomanometer. The initial systolic BP (SBP) and diastolic BP (DBP) values are determined by the standard auscultatory method. Auscultatory measurements are recorded by two independent observers (unaware of each other’s readings) using a mercury sphygmomanometer and a binaural stethoscope. The measurements are performed with the participants in the sitting position, with their forearms at heart level. Phase I and V Korotkoff sounds are used to assess the SBP and DBP, respectively. All auscultatory BP measurements are performed on the arm used for measurement with the CL-BP monitor. Two criteria are used in the evaluation.

Criterion 1.

The SBP and DBP differences in *n* individual paired measurements for all participants are within 5 ± 8 mmHg (mean ($\underset{\_}{{x}_{n}}$) ± standard deviation (Sn)) according to formulas (1) and (2):

1 $$\underset{\_}{{x}_{n}}=\frac{1}{n}\times {\sum }_{i=1}^{n}\left({p\left(T\right)}_{i}- {p\left(REF\right)}_{i}\right)$$

2 $${s}_{n}= \sqrt{\frac{1}{n-1}\times {\sum }_{i=1}^{n}{({x}_{i}- \underset{\_}{{x}_{n}})}^{2}}$$

 where *i* is the index for the individual participant, while *P(T)i-P(REF)i* is the difference between the ith paired CL-BP monitor BP value minus the reference BP value.

Criterion 2.

For the SBP and DBP for each of *m* participants, the standard deviation *s*m of the mean paired CL-BP monitor measurement and observers’ reading with the reference sphygmomanometer for each participant meet the criteria in Table S1, according to the following formulas:

3 $${s}_{m}= \sqrt{\frac{1}{m-1}\times {\sum }_{j=1}^{m}{({x}_{j}- \underset{\_}{{x}_{n}})}^{2}}$$

4 $${x}_{j}=\frac{1}{3}\times {\sum }_{k=1}^{3}\left({p\left(T\right)}_{k}- {p\left(REF\right)}_{k}\right)$$

 where $\underset{\_}{{x}_{n}}$ is the mean value of the differences in calculation (1); *m, j*, and x_j_ are the number of participants, the index for the individual participant, and the tested device error as calculated in (4), respectively; and *d, k*, and P(T)_k_-P(REF)_k_ are the number of tested participants, the k^th^ individual participant, and the difference between the paired CL-BP monitor’s BP values minus the reference BP values, respectively.

Accuracy measurements were performed 30 min after initialization. The obtained accuracy was defined as the device accuracy that met criteria 1 and 2.

Using the above criteria, we define the error of the developed device as error =  M(X_dut_) ± SD(X_dut_), where X_dut_ is the device under test; this means the accuracy of the developed device is tested with static error evaluation using an auscultation method.

#### Step 2. Long-term stability test

Long-term stability was evaluated by comparison with a medical-grade oscillometric automated sphygmomanometer. The reinitialization period was determined by the manufacturers’ instructions. In BP distribution, the rates of SBP measurements > 140 mmHg and < 100 mmHg were 20% and 5%, respectively; the rates of DBP measurements > 85 mmHg and < 60 mmHg were 20% and 5%, respectively.

The first initialization was conducted in accordance with the procedure in 3.3.1. Measurements began 10 min after the first initialization. Long-term stability was investigated over 1 month, in accordance with the following measurement protocol.

(1) Obtain a measurement using the CL-BP monitor,

(2) Obtain a measurement of reference BP using the oscillometric automated sphygmomanometer (UA-1200BLE, A & D, Japan) [51, 60],

(3) Wait at least 60 s,

(4) Repeat steps (1) to (3),

(5) Calculate the mean of the three CL-BP measurements and reference BP measurements. If the SBP and DBP values showed differences of > 12 and 8 mmHg, respectively, all data were excluded.

The measurements were performed twice on the first 3 days. Subsequently, two measurements were performed daily, in the morning and at night; one measurement was performed in the morning from days 4 to 10; and one measurement was performed every 10 days until day 30. This protocol was modified from ISODIS 81060-3.2.

#### Error determination

The mean and SD of the obtained data were calculated over the 1-month measurement interval. If the differences between the first initialization BP and first measured BP was more than 12 and 8 mmHg for SBP and DBP, respectively, the participant was excluded from the study. The mean and SD values differ from the values at rest because an automated sphygmomanometer was used, instead of an auscultatory method.

Because of error propagation, we propose another criterion. If an oscillometric reference sphygmomanometer is used as the standard, the error is the sum of the oscillometric automated sphygmomanometer error and the tested device error, as shown in Formula 5 with each error containing the mean ± SD.

5 $$M(({X_{OSC}}) \pm M({X_{dut}})) \pm \surd SD{({X_{OSC}})^2} + SD{({X_{dut}})^2}$$

The reference error (mean (X_osc_) ± SD (X_osc_)) is within 5 ± 8 mmHg; thus, the total device error was 10 ± 11.3 mmHg. A mean error of 10 mmHg is large and we thus propose use of the CL-BP monitor’s accuracy obtained at rest. If the CL-BP monitor’s accuracy is 4 ± 6 mmHg, the allowable error for long-term stability is 9 ± 10 mmHg. We obtained different long-term accuracies depending on the resting accuracy of the CL-BP monitor.

### Preliminary experiments

We evaluated accuracy at rest, accuracy in measuring BP changes, and long-term stability. The experiments were approved by the Ethics Committee of SAN-EI MEDISYS (#2019002SA) and written informed consent was obtained from all participants. Experiments were performed in accordance with the Declaration of Helsinki.

#### Step 1: accuracy test

Initial SBP and DBP values were determined by the standard auscultatory method. After performance of the initial BP measurements required to classify participants into BP categories, Checkme Pro (SAN-EI MEDISYS Japan) was continuously used by the participants until three successive measurements were achieved (i.e., the tested device was placed on the same arm used for validation). To ensure the short-term stability of device initialization, a 5-min rest period was allowed before validation measurements began. Three paired CL-BP monitor and auscultatory measurements were obtained, and the CL-BP monitor continuously measured BP throughout the procedure; BP values from the CL-BP monitor were determined by online analysis. A pause of 60 s was allowed between measurements to avoid the cuff inflation effect. The qualities of the ECG and PPG signals were checked periodically.

During the initialization session, data from the CL-BP monitors were transferred to a computer; BP values were calculated using BP estimation software. Validation BP values from the CL-BP monitor were obtained by calculating the mean SBP and DBP over 20–30 s good-quality segments. The obtained values were compared with the paired auscultatory BP measurements. Three comparison values were obtained, and the means were used as the initial values. During initialization, if the SBP and DBP values differed by more than 12 and 8 mmHg, respectively, all data were excluded.

The validation procedure was conducted in accordance with ISO 81060-2:2018 for the validation of BP measuring devices in adults. However, because of the characteristics of the CL-BP device, several adaptations were necessary. Three successive datasets were collected from 30 participants (18 men, 12 women; mean ± SD age, 40.2 ± 15.5 years [range, 28–71 years]). The BP distributions are shown in Table 2.

**Table 2:** BP distribution

	< 100	100–140	140–160	> 160
SBP	16.6% (5)	60% (18)	16.6% (5)	6.6% (2)
	< 60	60–85	85–100	> 100
DBP	10% (3)	56.6% (17)	23.3% (7)	10% (3)

Based on the preliminary study, we evaluated 30 participants.

#### Device and principle

Checkme Pro is an ambulatory vital sign-sensing device. The system consists of a finger PPG function that provides oxygen saturation measurement, as well as three embedded electrocardiography (ECG) leads with a control unit. The device is portable and equipped with a screen that displays beat-to-beat pulse waveform, ECG, and PTT changes. With the developed software, the PPG and ECG signals are transferred to the cloud via Bluetooth and the Internet. BP estimation is based on beat-to-beat determination of the PTT, calculated as the interval between the R-wave on ECG and the arrival of the corresponding pulse wave based on the PTT principle [61].

Using our BP estimation software, SBP and DBP were calculated based on the relationship between BP and PTT, where an increase in BP enhances arterial wall tension, thus increasing arterial wall stiffness [61–63]. Consequently, the pulse wave propagation velocity increases, leading to a reduction in the PTT. A linear model describing this relationship based on experimental data has been published.

6 $$SBP={SBP}_{0}- \frac{2}{\gamma {T}_{0}}\left(T-{T}_{0}\right)$$

7 $$DBP={SBP}_{0}- \frac{2}{\gamma {T}_{0}}\left(T-{T}_{0}\right)-\left({SBP}_{0}-{DBP}_{0}\right)-{\left(\frac{{T}_{0}}{T}\right)}^{2}$$

where T, SBP_0,_ DBP_0,_ T_0_, and γ are the PTT, initial SBP, initial DBP, initial PTT, and peripheral coefficient, respectively.

### Step 2: long-term stability test

Eighteen participants (10 men and 8 women; age, 46.7 ± 13.5 years [range, 28–71 years]) underwent this evaluation. Measurement of reference BP was performed using an oscillometric automated sphygmomanometer (UA-1200BLE; A & D, Japan) [51]. The protocol was as described in Sect. 3.2.2.

## Results

### Accuracy at rest

**Fig. 1:**
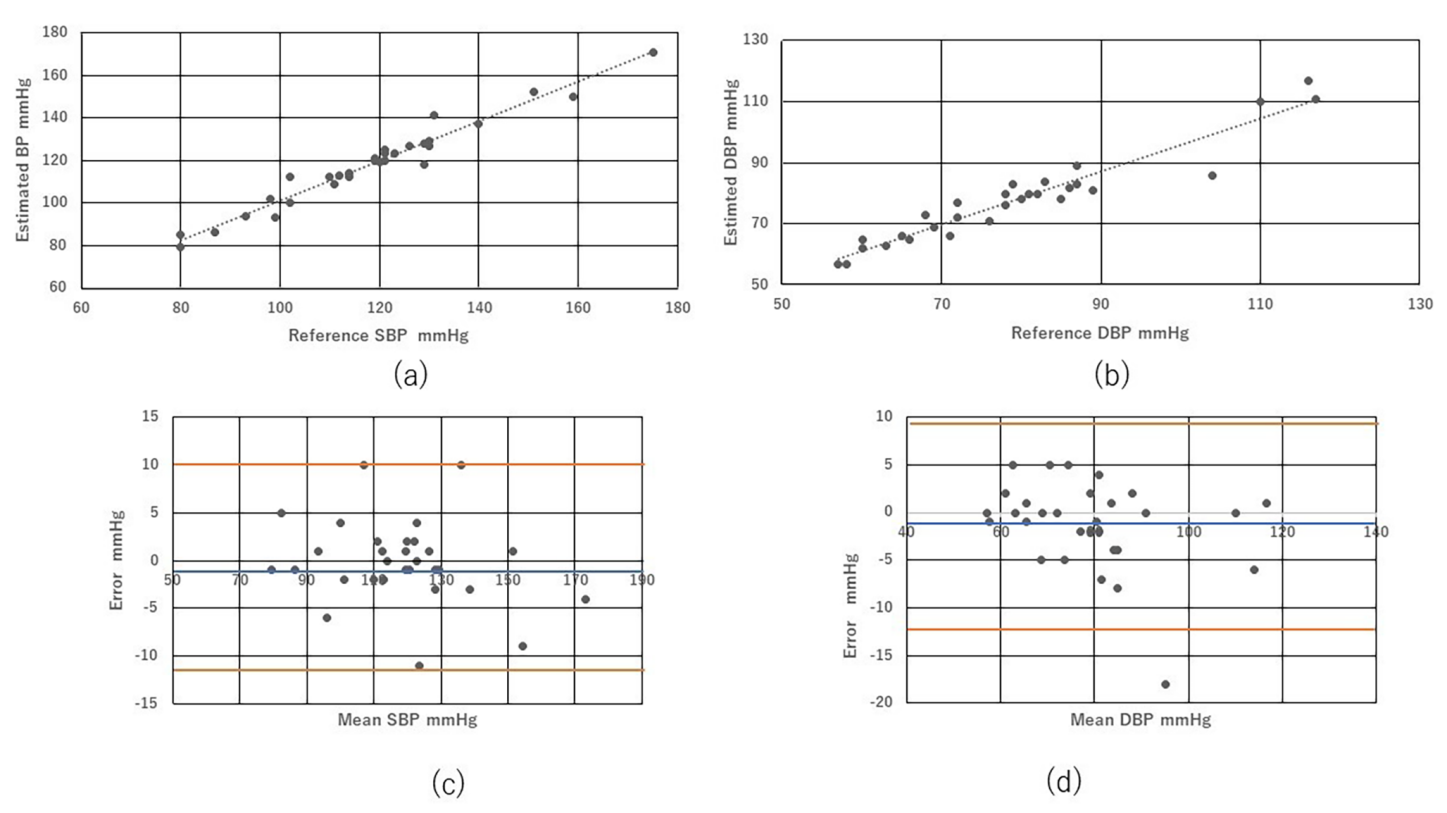
Scatterplots of SBP (a) and DBP (b) measured using an auscultation sphygmomanometer and estimated according to PTT. Bland–Altman plot of SBP (c) and DBP (d) values that met both criteria 1 and 2 of the standard (criterion 1, − 0.12 ± 5.49 and − 1.17 ± 5.06 mmHg, respectively; criterion 2, -0.12 ± 4.37 and − 1.17 ± 4.60 mmHg, respectively). The Bland-Altman plots did not show evidence of systematic error

The static accuracy was reasonably good and was maintained within ± 5 ± 8 mmHg (Table 3). The correlations of SBP and DBP are shown in Fig. 1(a) and (b), respectively. Bland-Altman plots of SBP and DBP by the CL-BP monitor and standard sphygmomanometer are shown in Fig. 1(c) and (d), respectively.

**Table 3:** Static accuracy

		Results		Permissible ranges		Judgment
		SBP mmHg	DBP mmHg	SBP mmHg	DBP mmHg	
Criterion 1	Mean	-0.12	-1.17	± 5.0	± 5.0	Pass
	SD	5.49	5.06	8.0	8.0	Pass
Criterion 2	Mean	-0.12	-1.17	± 5.0	± 5.0	Pass
	SD	4.37	4.60	< 6.95	< 6.73	Pass

### Long-term stability

Four of 18 participants showed a 12-mmHg difference between initialization and the first measurement; they were excluded from the analysis. Static accuracies were − 0.12 ± 5.49 mmHg for SBP and − 1.17 ± 5.06 mmHg for DBP. The measurements obtained using the oscillometric automated sphygmomanometer were within 5 ± 8 mmHg. Based on the propagation of error, the accuracy criteria of the long-term stability of SBP and DBP measurements obtained using the oscillometric automated sphygmomanometer were − 5.12 ± 9.70 mmHg and − 6.17 ± 9.48 mmHg, respectively. The long-term accuracies of SBP and DBP were − 3.38 ± 7.1 mmHg and − 1.38 ± 5.4 mmHg, respectively; these values met the criteria (Supplementary Table S2).

**Fig. 2:**
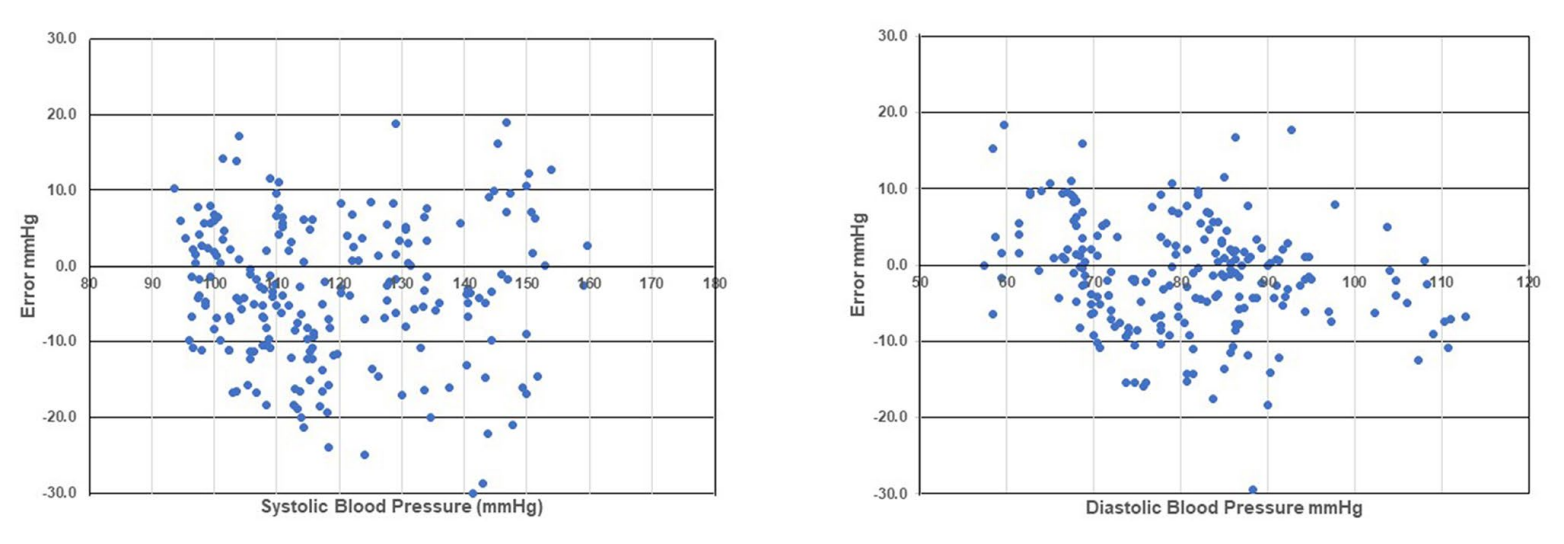
Numbers of absolute BP differences (test BP minus mean versus reference BP readings)

The numbers of absolute BP differences (test BP minus mean *versus* reference BP readings), within 5, 10, and 15 mmHg for SBP and DBP, are presented in Fig. 2, along with standardized Bland–Altman scatterplots. A device is considered acceptable if its estimated probability of a tolerable error (≤ 10 mmHg) is at least 85%. Table 4 shows the ratio of the number of absolute BP differences (test BP minus mean *versus* reference BP readings) to the total number of data points.

**Table 4:** Ratio of the number of absolute BP differences to the total number of data points

Error range	± 10 mmHg	± 15 mmHg	± 20 mmHg
Number of absolute BP differences (test BP minus mean *versus* reference BP readings)% (n = 15) SBP/DBP	85.2%/91.9%	88.6%/94.8%	96.2%/99.5%

## Discussion

In the World Health Organization report on hypertension, self-management of BP monitoring was considered an important function [36, 45, 52, 53, 64, 65]. Therefore, simple and reliable BP monitors are needed, particularly in low-income countries. To maintain device accuracy and validity, standards are required. Here, we discuss standardization of CL-BP monitors, with evaluations of static error, and long-term stability.

### Static error and long-term stability

The static accuracy of CL-BP monitors is identical to the static accuracy of cuff-based sphygmomanometers. Concerning the auscultation reference method, the requirements must be discussed. Our sample size was small, but the BP distribution percentage in the standard protocol was covered. A comprehensive experiment requires 85 subjects for precise measurement. The relationship between sample size and experimental period should be considered.

Long-term validation is important in evaluations of CL-BP monitors. For long-term evaluations, we propose a new accuracy criterion that encompasses the number of participants and a standard device.

In both research protocols and manufacturer reviews, participant recruitment is an important consideration. If a device does not require reinitialization within approximately 1 month, the comparison study must be of 1-month duration. Because of the difficulty of recruitment, we enrolled fewer than 20 participants, some of whom did not meet the inclusion criteria. The recruitment of participants with high BP, particularly a high DBP, was difficult and constituted most of the additional screened participants; this is reflected in the overall distribution, as shown in the DBP plot, where most of the points are < 115 mmHg. Because the daily BP changes are close to the distribution in each range in DBP, the distribution conditions are fulfilled. A tolerable error of ≤ 10 mmHg (using an individual’s mean BP readings *versus* a reference BP measurement method) and an estimated probability of error of at least 85% are acceptable as a compromise, considering that performance is similar to currently available BP monitors [66–69].

Although opinions are divided, the use of a medically approved oscillometric sphygmomanometer is easy, convenient, and practical for long-term stability tests. Although the auscultation method is more accurate and complete, the method is complicated and requires two observers. The development of semiconductor devices enables the production of precise, reliable oscillometric sphygmomanometers for the market. Thus, we propose the criterion based on error propagation shown in Formula 6.

The absolute error in the result is equal to the sum of the absolute error in the observed quantities. We propose a simple and practical solution to challenges concerning the use of oscillometric sphygmomanometers.

A few articles have discussed the long-term stability of CL-BP monitors [27]. They followed the original auscultation protocol and the results met the criteria, but the patient was visited only 1 day per week. That type of CL-BP is calibrated to the individual, which seems like a large study burden. Mostly, those BP values were compared with cuff-based sphygmomanometers [43, 66]. The Aktiia group also attempted to monitor patients for 2 or 3 months without reinitialization [70].

### Standard

We determined intermittent accuracy using an automated sphygmomanometer under ISO81060-2. The long-term stability accuracy standard of 5 ± 8 mmHg was not satisfied in this study. If our method is to be considered acceptable for long-term stability studies aimed at improving accuracy, standardization is required. The cuffless blood pressure monitor’s accuracy should be quantified in large studies involving subjects with varying blood pressure values. The European Society of Hypertension International Protocol (EHS-IP) reduced the recommended sample size from 85 to 33. However, the regulatory authorities in the USA and Europe require validation with the longer, more robust 85-subject protocols, and all manufacturers selling products on these markets need to use the protocol described in the ANSI/AAMI/ISO 81060-2 standards (which are recommended by most clinicians).

In addition, individual laboratory results should be closely monitored; often, accuracy requirements are not satisfied [71]. Thus, standardized testing protocols and guidelines should be established through collaboration among regulatory authorities, research institutes, medical professionals, and manufacturers [72].

The difference between the ISO/FDIS 81060-3 and our proposal is the use of a standard sphygmomanometer, either auscultatory or oscillometric. Moreover, the accuracy of the long-term stability mainly involves direct BP monitoring. The volume clamp method in the operating room and ICU is the target of ISO/FDIS 81060-3. Even a comparison study involving direct BP monitoring for more than 10 days is a heavy burden on medical staff.

Moreover, the intra-correlation is used in the standard. This is reasonable and individuals have different daily activities. Without individual calibration, the intra-correlation coefficient is much more reliable than the standard statistics we used.

### Dynamic response

A beat-to-beat evaluation can be conducted if a direct arterial BP monitor is used as a standard device. However, manufacturers need time and resources to conduct such evaluations. Additionally, several countries do not allow direct arterial BP monitoring in healthy individuals.

At present, if manufacturers would like to distribute a device for use in continuous clinical practice (e.g., for nocturnal BP monitoring and BP monitoring during anesthesia), they must perform a comparison study with direct arterial BP monitoring. Another option involves the use of the volume clamp method as a standard, in accordance with ISO/FDIS 81060-3 Noninvasive Sphygmomanometers-Part 3: Clinical Investigation of the Continuous Automated Measurement Type. Therefore, the definition of a beat-to-beat evaluation in the proposed procedure was difficult, and further discussions of beat-to-beat evaluations are needed. New ideas for standardization protocols must be considered for the evaluation of dynamic changes.

### Regulation of medical devices

From a regulatory point of view, new devices must be highly precise. Embedded devices and medical software are used to avoid misdiagnoses [73, 74]. Regarding the regulatory framework, guidance on the predetermined methods through which software learns over time is required, as is a patient-centered approach. Medical device operation must be clear to patients, and improved machine-learning algorithms and real-world performance-monitoring methods are needed. In the era of the COVID-19 pandemic, new medical device regulation is more important than addressing errors of approved devices [75]. Medical professionals should exercise caution when recommending over-the-counter medical devices care. Regulatory issues require further debate.

## Conclusion

We proposed a protocol for evaluating the static accuracy and long-term stability of the CL-BP monitoring system. Due to experimental protocols, we were unable to perform a comparative study to evaluate the dynamic response. Our long-term validation study involving 15 participants did not meet the minimal validation criteria. Our revised proposal includes a new evaluation process for long-term stability assessments. Our study population size of 15 was small, but our results provide a roadmap for popularizing cuffless blood pressure monitors for screening purposes, and potentially other over-the-counter healthcare devices. Collectively, our findings will promote the formulation of a new standard.

## Electronic supplementary material

Below is the link to the electronic supplementary material.

Supplementary Material 1

## Data Availability

All data analyzed during the current study are available from the corresponding author on reasonable request.
