# Supplementary material for: Long-term stability of over-the-counter cuffless blood pressure monitors: a proposal
Source: Health Technol (Berl). 2023 Jan 23;13(1):53–63. doi: 10.1007/s12553-023-00726-6 (PMC9870659; doi:10.1007/s12553-023-00726-6)
Supplement: Supplementary file 1 — Supplementary Material 1 [file 12553_2023_726_MOESM1_ESM.docx]

Supplementary Table S1 Average subject data acceptance (criterion 2) in mmHg

| $\bar{x_{n}}$ | Maximum permissible standard deviation, S_m_, as function of $\bar{x_{n}}$ (mmHg) | | | | | | | | | |
| --- | --- | --- | --- | --- | --- | --- | --- | --- | --- | --- |
|  | 0 | 0.1 | 0.2 | 0.3 | 0.4 | 0.5 | 0.6 | 0.7 | 0.8 | 0.9 |
| ±0. | 6.95 | 6.95 | 6.95 | 6.95 | 6.93 | 6.92 | 6.91 | 6.9 | 6.89 | 6.88 |
| ±1. | 6.87 | 6.86 | 6.84 | 6.82 | 6.8 | 6.78 | 6.76 | 6.73 | 6.71 | 6.68 |
| ±2. | 6.65 | 6.62 | 6.58 | 6.55 | 6.51 | 6.47 | 6.43 | 6.39 | 6.34 | 6.3 |
| ±3. | 6.25 | 6.2 | 6.14 | 6.09 | 6.03 | 5.97 | 5.89 | 5.83 | 5.77 | 5.7 |
| ±4. | 5.64 | 5.56 | 5.49 | 5.41 | 5.33 | 5.25 | 5.16 | 5.08 | 5.01 | 4.9 |
| ±5. | 4.79 | — | — | — | — | — | — | — | — | — |
| Example for mean of ±4.2 mmHg; the maximum permissible standard deviation is 5.49 mmHg | | | | | | | | | | |

Supplementary Table S2 One-month long-term stability for each individual

|  | Systolic blood pressure | | Diastolic blood pressure | |
| --- | --- | --- | --- | --- |
| Subject number | Mean (mmHg) | SD (mmHg) | Mean (mmHg) | SD (mmHg) |
| 1 | 7.96 | 6.11 | 4.32 | 4.09 |
| 2 | 0.51 | 8.63 | -2.27 | 5.48 |
| 3 | 3.31 | 8.20 | 2.84 | 5.66 |
| 4 | -0.99 | 4.84 | -0.64 | 2.20 |
| 5 | -12.15 | 8.03 | -2.16 | 7.66 |
| 6 | -3.66 | 7.85 | 5.79 | 6.77 |
| 7 | 2.25 | 5.07 | 4.95 | 4.53 |
| 8 | -6.34 | 9.38 | -6.41 | 7.22 |
| 9 | -7.86 | 7.20 | -4.89 | 4.69 |
| 10 | -7.86 | 4.86 | -3.48 | 5.94 |
| 11 | -8.15 | 6.34 | -7.25 | 3.81 |
| 12 | -5.46 | 5.16 | -3.64 | 5.12 |
| 13 | -0.11 | 8.97 | -0.96 | 6.36 |
| 14 | -8.74 | 8.76 | -5.50 | 7.04 |
| Mean±SD | -3.38 | 7.10 | -1.38 | 5.47 |
